# Pectic Bee Pollen Polysaccharide from *Rosa rugosa* Alleviates Diet-Induced Hepatic Steatosis and Insulin Resistance via Induction of AMPK/mTOR-Mediated Autophagy

**DOI:** 10.3390/molecules22050699

**Published:** 2017-04-28

**Authors:** Xinzhi Li, Haiquan Gong, Siwen Yang, Lulu Yang, Yuying Fan, Yifa Zhou

**Affiliations:** School of Life Sciences, Northeast Normal University, Changchun 130024, China; lixz329@nenu.edu.cn (X.L.); gonghq106@nenu.edu.cn (H.G.); yangsw776@nenu.edu.cn (S.Y.); yangll132@nenu.edu.cn (L.Y.)

**Keywords:** bee pollen, pectic polysaccharide, hepatic steatosis, insulin resistance, autophagy

## Abstract

Despite it is used as a nutraceutical against diabetes and obesity, the mechanism of action of bee pollen is still unclear. Pectic bee pollen polysaccharide (RBPP-P) was isolated from *Rosa rugosa*, and its structure was characterized by ^13^C-NMR and Fourier transform-infrared spectroscopy (FT-IR). Using high glucose and fatty acids-treated HepG2 cells and high fat diet (HFD)-induced obesity mice, we detected its effect on insulin function and lipid metabolism based on autophagy. RBPP-P contained arabinogalactan, rhamnogalacturonan I, and homogalacturonan domains. In vivo studies demonstrated that RBPP-P markedly ameliorated insulin resistance, glucose intolerance, and liver steatosis in obese mice. The suppressive effects of RBPP-P on liver steatosis and triglyceride content were mediated by increased autophagy and lipase expression in liver. In AMPK knockdown cells (*prkaa 1/2^−/−^* MEF) and HFD-fed mice tissues (liver, gonadal white adipose, and inguinal white adipose), RBPP-P enhanced autophagy in AMPK/mTOR-dependent way in liver, but not in adipose tissue. These findings demonstrated that bee pollen polysaccharide alleviated liver steatosis and insulin resistance by promoting autophagy via an AMPK/mTOR-mediated signaling pathway, suggesting that RBPP-P could be a novel therapeutic agent used for the treatment of obesity and diabetes.

## 1. Introduction

Bee pollen is a mass of pollen grains in the hive after storage and fermentation. Because bees collect the most abundant nutrients on a variety of stamens, bee pollen is also known as “essence of flowers” [1,2]. Bee pollen has been widely used as a nutraceutical due to its unverified effects against obesity and diabetes. Accumulated evidence has indicated that bee pollen is mainly composed of carbohydrate, amino acid, lipid, and minerals [1,3,4]. Among them, the polysaccharide is the main and non-toxic active component [3,5,6]. It has been reported that bee pollen polysaccharide from *Crataegus pinnatifida* increased phagocytic rates of macrophage and stimulated spleen cell proliferation [3]. However, the effects of bee pollen polysaccharide on obesity and diabetes and the underlying mechanisms have not been fully investigated.

Obesity is prevalent metabolic disorder which results from an imbalance between lipogenesis and lipolysis. Obesity is often accompanied by liver steatosis and insulin resistance. Liver steatosis is characterized by excess triglyceride accumulation in liver, leading to hepatocytes being less sensitive to insulin. Autophagy is a critical pathway for the degradation of damaged intracellular components, including lipids. Impaired autophagic function may promote the development of liver steatosis. 

In the present study, bee pollen polysaccharide from *Rosa rugosa* (RBPP) was isolated and purified into neutral polysaccharide fraction (RBPP-N) and pectic polysaccharide fraction (RBPP-P); we characterized the structural features of RBPP-P by ^13^C-NMR and Fourier transform-infrared spectroscopy (FT-IR). RBPP-P markedly ameliorated insulin resistance in vivo and in vitro, and improved glucose intolerance and liver steatosis in mice with high fat diet (HFD)-induced obesity. Furthermore, RBPP-P enhanced autophagy in an AMPK/mTOR-dependent manner in liver, but not in adipose tissue. These findings demonstrated that RBPP-P alleviated liver steatosis and insulin resistance by promoting autophagy via an AMPK/mTOR-mediated signaling pathway.

## 2. Results

### 2.1. Preparation and Characterization of Bee Pollen Polysaccharides

**Figure 1:**
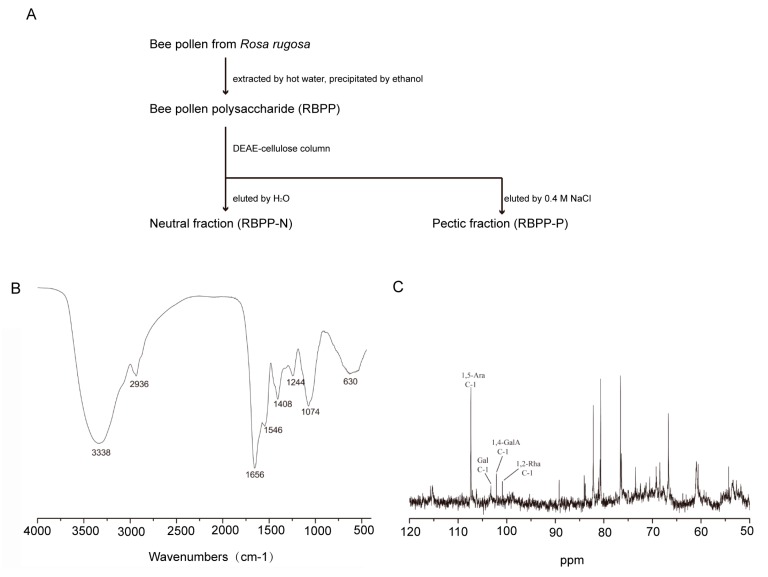
Preparation and characterization of bee pollen polysaccharides. (**A**) The procedure used to prepare the sub-fractions of bee pollen polysaccharide from *Rosa rugosa* (RBPP) including RBPP-P and RBPP-N (-pectic polysaccharide and neutral polysaccharide fractions of RBPP, respectively); (**B**) Analysis of the chemical structure of RBPP-P using Fourier transform-infrared spectroscopy (FT-IR); (**C**) Analysis of chemical structure of RBPP-P using ^13^C-NMR.

Bee pollen polysaccharide (RBPP) was obtained from *Rosa rugosa* by hot water extraction and ethanol precipitation. It was then separated by DEAE-Cellulose column, eluted with water and 0.4 M NaCl, respectively. Two sub-fractions (RBPP-N and RBPP-P) were obtained (Figure 1A). The yield of RBPP-P (35.8%) was higher than that of RBPP-N (23.4%). Their monosaccharide compositions are listed in Table 1. RBPP-N was a neutral fraction composed of Glc (glucose, 35.0%), Ara (arabinose, 37.8%), and Gal (galactose, 27.2%); its possible structure might be glucan and arabinogalactan (AG). RBPP-P contained Ara (51.8%), Gal (21.0%), GalA (galacturonic acid, 18.9%), and Rha (rhamnose, 5.4%), which were typical monosaccharides for pectin.

In the FT-IR spectrum of RBPP-P (Figure 1B), absorption at 3338 cm^−1^ was the characteristic of the hydroxyl group. The characteristic absorption at 1074 cm^−1^ was ascribed to α-type glucopyranose linkage in the polysaccharide. The characteristic absorption at 2936 cm^−1^ indicated the presence of methyl or methylene. The band from 1656 cm^−1^ to 1244 cm^−1^ also demonstrated the existence of a free carboxyl of uronic acid hydroxyl bending vibration absorption peak.

The structural feature of RBPP-P was further analyzed by ^13^C-NMR (Figure 1C). As can be seen, the anomeric signal at 108.3 ppm belongs to C-1 of α-1,5-Ara*f* (furan arabinose), the signal at 104.5 ppm belongs to Gal; both had a large proportion, suggesting the existence of arabinogalactan (AG) fragments. The signals at 102.5 and 102.8 ppm belong to C-1 of α-1,4-Gal*p*A (pyranose galacturonic acid) and α-1,2-Rha*p* (pyranose rhamnose), respectively, indicating the existence of small amounts of rhamnogalacturonan I (RG-I) and homogalacturonan (HG) domains. 

### 2.2. RBPP-P Increased Insulin Sensitivity in HepG2 Cells Treated with High Glucose and Fatty Acids

**Figure 2:**
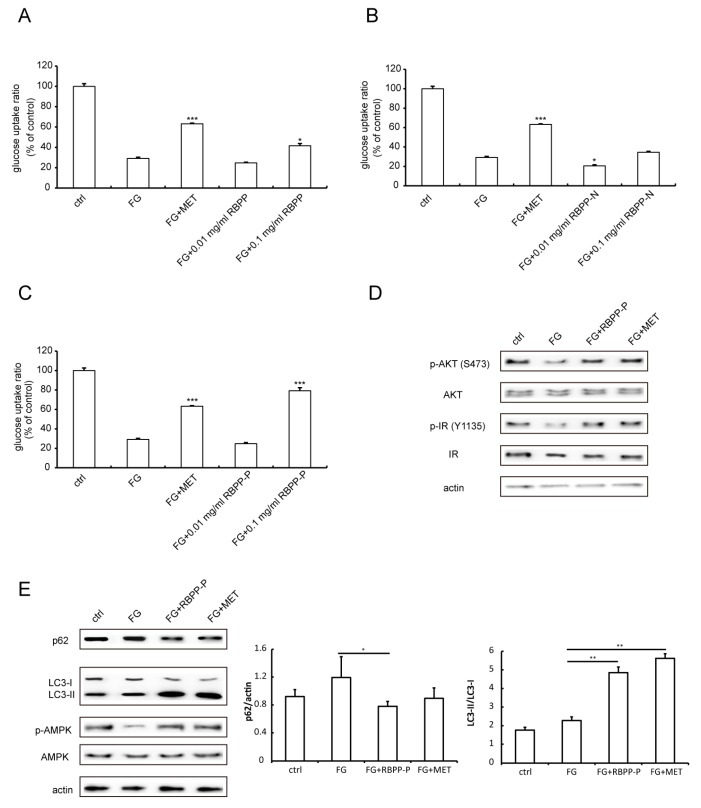
Effects of RBPP, RBPP-P, and RBPP-N on insulin resistance in vitro. HepG2 cells were induced by FG-conditioned medium (25 mM high glucose and 0.5 mM fatty acids); (**A**) Effects of RBPP; (**B**) Effects of RBPP-N; (**C**) Effects of RBPP-P; (**D**) RBPP-P-enhanced insulin signaling. Phosphorylation of Insulin receptor (IR) and AKT in HepG2 cells incubated with or without insulin (50 nM, 10 min) was detected by Western blot; (**E**) Western blot analysis of autophagy by detecting p62, LC3-II levels and phosphorylation of AMPK in HepG2 cells treated with control, FG, FG plus RBPP-P (0.1 mg/mL), or FG plus MET (20 μM). Results represent mean ± s.d. (*n* = 4 incubations in each group) * *p* < 0.05; ** *p* < 0.01; *** *p* < 0.001 as compared to FG.

To screen the active components’ contribution to the anti-obesity and anti-diabetic activity of bee pollen, we examined the protective effects of bee pollen polysaccharides on insulin resistance induced by high glucose and fatty acids (FG) in HepG2 cells. As shown in Figure 2A–C, glucose uptake was significantly reduced by FG treatment as compared with control, indicating that FG induced insulin resistance in HepG2 cells. Metformin (MET, 20 μM)—an anti-diabetic drug—significantly attenuated FG-induced insulin resistance by 60%. However, treatment with RBPP (0.01 mg/mL), RBPP-N (0.01 mg/mL or 0.1 mg/mL), or RBPP-P (0.01 mg/mL) did not significantly alter FG-induced insulin resistance (Figure 2A–C). More interestingly, high doses of RBPP (0.1 mg/mL) and RBPP-P (0.1 mg/mL) ameliorated FG-induced insulin resistance; especially, the attenuation of RBPP-P dramatically reached 70% (Figure 2A,C). Therefore, the following experiments were focused on RBPP-P.

We next examined the molecular mechanism underlying the antagonizing effect of RBPP-P on FG-induced insulin resistance in HepG2 cells. The results showed that RBPP-P or MET treatment enhanced insulin-stimulated phosphorylation of insulin receptor (IR) and AKT, a downstream signaling molecule of IR (Figure 2D). In summary, the RBPP-P-induced elevation of insulin sensitivity could be mediated by an IR/AKT signaling pathway. 

Previous studies demonstrated that autophagy is essential to insulin function in mammalian cells [7]. The p62 protein is one of the selective substrates for autophagy, as well as a scaffold in autophagosomes. The LIR (LC3-interacting region) of p62 is responsible for binding autophagy regulator Atg8/LC3. During autophagy, cytosolic LC3-I is conjugated to phosphatidylethanolamine (PE) to become LC3-II, which is localized in the autolysosomal lumen [8]. Thus, degradation of p62 and LC3 conversion (LC3-I to LC3-II) reflect the progression of autophagy. Herein, we next examined the effect of RBPP-P on the autophagy of HepG2 cells by detecting p62 degradation and LC3-II conversion using Western blots. The results demonstrated that the treatment of cells with FG did not significantly alter autophagy. However, the treatment of cells with FG plus RBPP-P or MET dramatically enhanced autophagy, suggesting that RBPP-P activated autophagy (Figure 2E). We also examined the effect of RBPP-P on AMPK activity, demonstrating that RBPP-P treatment significantly increased phosphorylation of AMPK, comparable with the effect of MET (Figure 2E). Taken together, autophagy induction of RBPP-P could be mediated by AMPK activation in HepG2 cells.

### 2.3. RBPP-P Ameliorated Insulin Resistance and Glucose Intolerance in HFD-Fed Mice

**Figure 3:**
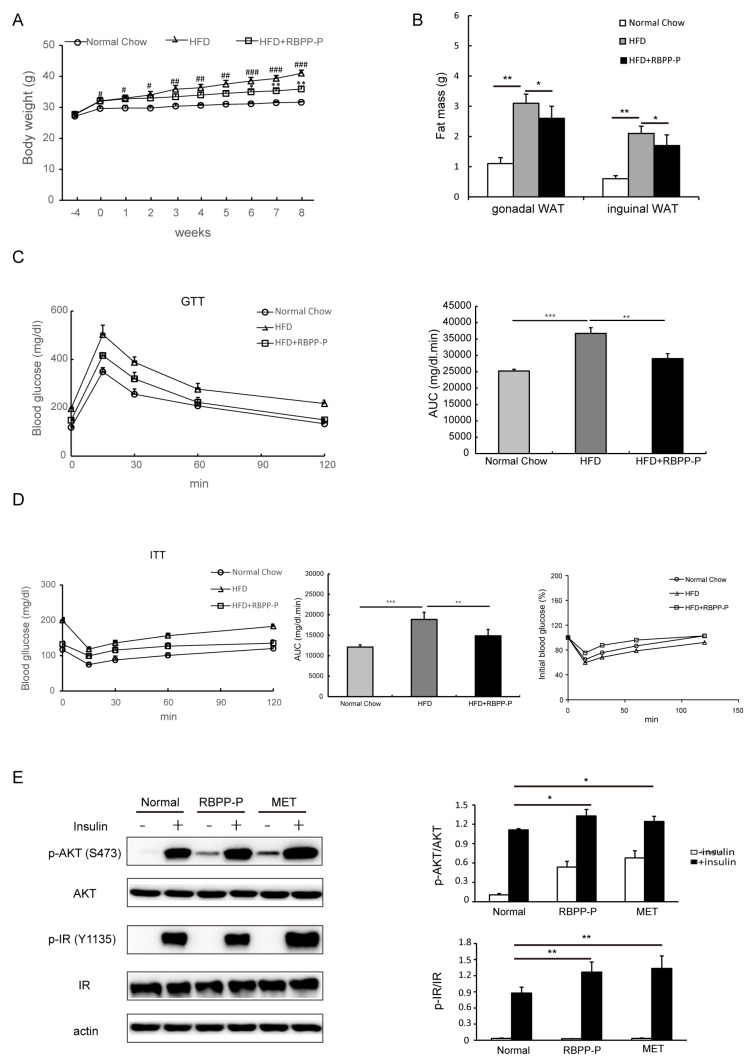
Effect of RBPP-P on insulin resistance in mice with high-fat diet (HFD)-induced obesity. Mice were pretreated with high fat diet (HFD) for 4 weeks, followed by treatment with normal chow, HFD, or HFD plus RBPP-P (20 mg/kg, intraperitoneal injection, once daily) for 8 weeks. (**A**) Effects of HFD and RBPP-P on body weight of mice; (**B**) Effect of HFD and RBPP-P on the mass of adipose tissues; (**C**) Effects of HFD and RBPP-P on glucose tolerance (left), and calculation of area under the curve (AUC) from glucose tolerance tests (GTT, right); (**D**) Effects of HFD and RBPP-P on insulin tolerance (left), calculation of AUC from insulin tolerance test (ITT, middle), and percentage of initial blood glucose level (right); (**E**) Effects of RBPP-P and metformin (MET) on the phosphorylation of insulin receptor (IR) and AKT in hepatocytes cultured from mice. Results represent mean ± s.d. (*n* = 8 mice in each group). # *p* < 0.05; ## *p* < 0.01; ### *p* < 0.001 as compared with mice fed with normal chow. * *p* < 0.05; ** *p* < 0.01 as compared with mice fed with HFD.

To further determine the effect of RBPP-P on insulin resistance, HFD-induced type 2 diabetes mice were treated with RBPP-P (20 mg/kg body weight, intraperitoneal injection, once daily) for 8 weeks. We examined the influence of sole RBPP-P administration on mice (Figure S1). The three physiological indicators of RBPP-P-treated mice, including body weight (Figure S1A), fasting blood glucose (Figure S1B), and serum alanine transaminase (ALT) level (Figure S1C) were similar to the control mice, indicating that RBPP-P had no side effects on mice physiological activities within 8 weeks of administration. Body weight was increased consistently in 4 weeks HFD, and kept increasing during 8 weeks’ treatment with HFD as compared with mice treated with normal chow. RBPP-P treatment significantly attenuated HFD-induced increases in body weight by 50% as compared with mice treated with HFD only (Figure 3A). Similarly, HFD-induced increases in fat mass were also attenuated by RBPP-P treatment for 8 weeks (Figure 3B). Tumor necrosis factor α (TNF-α) and interleukins (IL-6) are the major regulators of adipose tissue metabolism [9,10], so the contents of TNF-α and IL-6 were also examined in gonadal white adipose tissue and inguinal white adipose tissue. RBPP-P treatment significantly decreased HFD-induced increases in contents of these cytokines (Figure S2).

In addition, the glucose tolerance and insulin tolerance were also examined in the mice treated with normal chow, HFD, and HFD plus RBPP-P (20 mg/kg body weight, intraperitoneal injection, once daily) for 8 weeks. HFD treatment significantly induced glucose intolerance and insulin resistance, but RBPP-P treatment dramatically attenuated HFD-induced glucose intolerance and insulin resistance (Figure 3C,D). To further confirm that RBPP-P ameliorated HFD-induced insulin resistance, we examined the IR signaling in primary hepatocytes cultured from mice. Incubation of cells with insulin (50 nM, 10 min) significantly increased the phosphorylation of both IR and AKT. Treatment with RBPP-P or MET significantly increased basal and insulin-induced phosphorylation of IR and AKT in cultured hepatocytes (Figure 3E).

### 2.4. RBPP-P Lowered Serum Triglyceride, Low-Density Lipoprotein-Cholesterol, and Insulin Levels in HFD-Fed Mice

**Figure 4:**
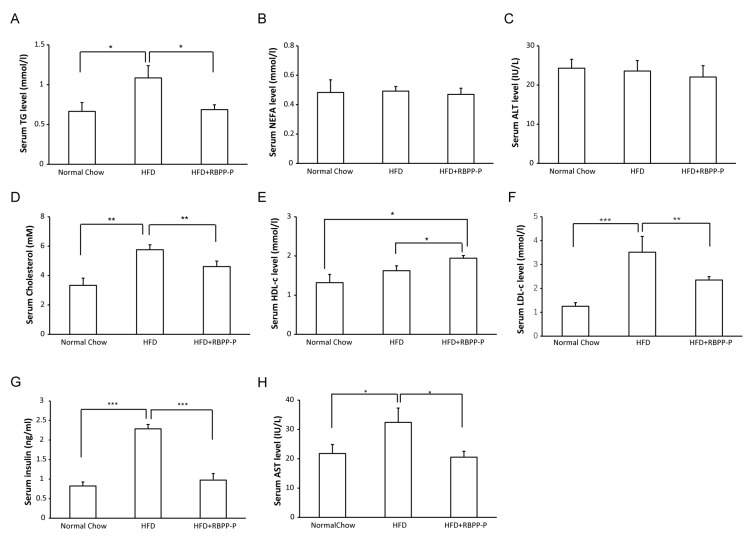
Metabolic profiles of HFD-fed mice treated with RBPP-P. (**A**) triglyceride (TG); (**B**) non-esterified free fatty acid (NEFA); (**C**) alanine transaminase (ALT); (**D**) cholesterol; (**E**) high-density lipoprotein cholesterol (HDL-c); (**F**) low-density lipoprotein cholesterol (LDL-c); (**G**) insulin; (**H**) aspartate transaminase (AST). Results represent mean ± s.d. (*n* = 8 mice in each group). * *p* < 0.05; ** *p* < 0.01; *** *p* < 0.001.

To investigate effect of RBPP-P on lipid metabolism, we measured the plasma levels of triglyceride (TG), non-esterified free fatty acid (NEFA), total cholesterol, high-density lipoprotein (HDL)-cholesterol, low-density lipoprotein (LDL)-cholesterol, alanine transaminase (ALT), and aspartate transaminase (AST). HFD treatment did not seem to alter serum ALT levels (Figure 4C), but markedly elevated AST levels (Figure 4H), suggesting that HFD might cause liver damage. RBPP-P treatment (20 mg/kg body weight, intraperitoneal injection, once daily) lowered AST levels, suggesting that RBPP-P had a protective effect on the liver (Figure 4C,H). HFD treatment significantly increased serum TG levels as compared with mice with normal chow, and HFD-induced elevation in TG levels were attenuated by 36.3% in mice treated with RBPP-P (20 mg/kg body weight, intraperitoneal injection, once daily) for 8 weeks (Figure 4A). Cholesterol levels in HFD-fed mice were significantly elevated as compared with the normal chow mice. RBPP-P treatment dramatically attenuated HFD-induced elevation in total cholesterol and LDL-cholesterol levels (Figure 4D,F). More interestingly, RBPP-P significantly raised serum HDL-cholesterol, a beneficial plasma lipid (Figure 4E). However, neither HFD nor RBPP-P altered serum NEFA level (Figure 4B). Taken together, RBPP-P ameliorated dyslipidemia in mice with diet-induced obesity. 

Accumulated evidence has demonstrated that C57BL/6J mice fed with high fat diet show stable hyperglycemia and progressively increased hyperinsulinemia, indicating progressive worsening of insulin resistance [11]. As shown in Figure 4G, the insulin in HFD-fed mice was significantly higher than that of the mice fed with normal chow, indicating that high fat diet could induce hyperinsulinemia and insulin resistance. RBPP-P treatment markedly attenuated HFD-induced hyperinsulinemia.

### 2.5. RBPP-P Accelerated Hepatic Lipolysis in HFD-Fed Mice

**Figure 5:**
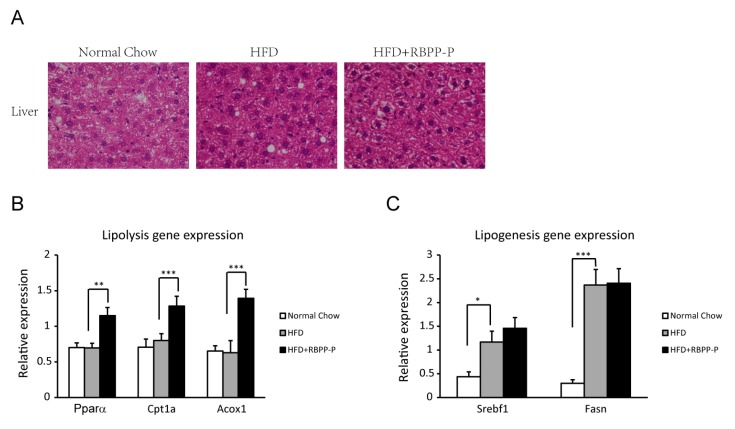
Effects of RBPP-P and HFD on liver lipid metabolism in mice. (**A**) Effects of HFD and RBPP-P on the number of lipid droplets in liver; (**B**) Expressions of lipolysis-related genes in liver (*Pparα*: peroxisome proliferator-activated receptor; *Cpt1a*: carnitine palmitoyl transferase 1a; *Acox1*: acyl-coenzyme A oxidase 1); (**C**) Expressions of lipogenesis-related genes in liver (*Srebf1*: sterol regulatory element binding transcription factor 1; *Fasn*: fatty acid synthase). Results represent mean ± s.d. (*n* = 8 mice in each group). * *p* < 0.05; ** *p* < 0.01; *** *p* < 0.001.

To determine the effect of RBPP-P on lipid metabolism in liver, the lipid droplets and lipid-metabolism-related enzyme expression were examined in liver of mice treated with normal chow, HFD, and HFD plus RBPP-P using hematoxylin and eosin (H&E) staining and quantitative real-time PCR (RT-qPCR). HFD significantly increased lipid droplet number in liver, and RBPP-P treatment dramatically attenuated HFD-induced increase in lipid droplet amount in liver (Figure 5A). In addition, the expressions of hepatic lipolytic genes, including peroxisome proliferator-activated receptor (*Pparα*), carnitine palmitoyl transferase 1a (*Cpt1a*), and acyl-coenzyme A oxidase 1 (*Acox1*), were significantly elevated in mice fed with HFD plus RBPP-P (Figure 5B). In contrast, the expressions of lipogenic genes, including sterol regulatory element binding transcription factor 1 (*Srebf1*) and fatty acid synthase (*Fasn*), were comparable between HFD-fed mice and mice fed with HFD plus RBPP-P (Figure 5C). These data suggested that RBPP-P inhibited hepatic steatosis mainly by increasing the expressions of lipolytic genes.

### 2.6. RBPP-P Attenuated HFD-Induced Inhibition in Hepatic Autophagy via AMPK/mTOR-Dependent Pathway in Mice 

**Figure 6:**
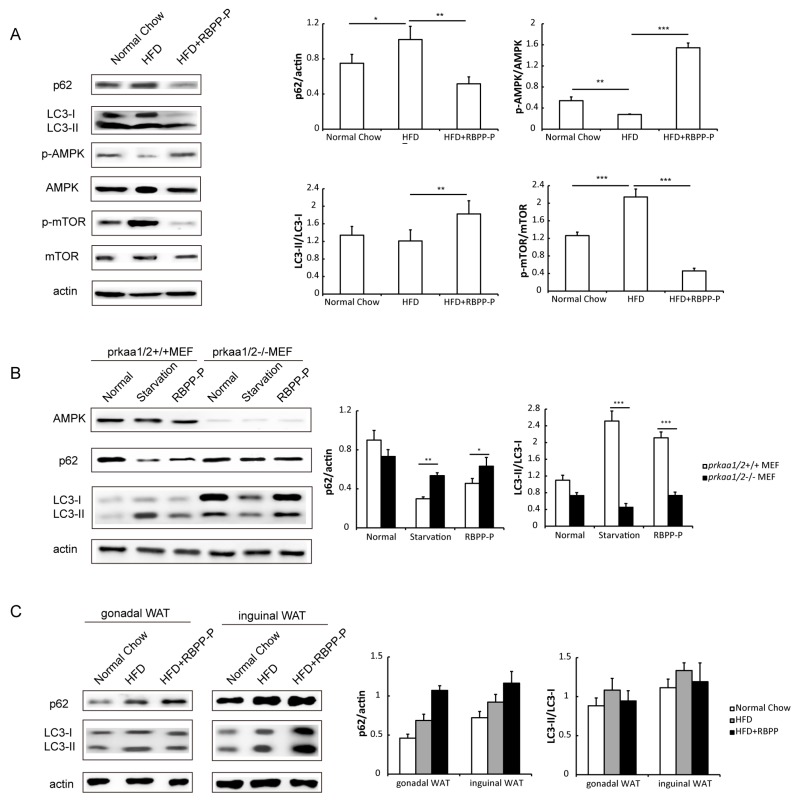
RBPP-P attenuated HFD-induced inhibition in hepatic autophagy in mice. (**A**) Effects of RBPP-P and HFD on AMPK/mTOR signaling pathway and autophagy-related proteins expressions analyzed using Western blots in the liver of mice; (**B**) Effects of RBPP-P and starvation on p62 and LC3 protein levels in control and AMPK knockdown cells; (**C**) Effects of HFD and RBPP-P on p62 and LC3 protein levels in adipose tissues of mice treated with normal chow, HFD, or HFD plus BRPP-P for 8 weeks. Results represent mean ± s.d. (*n* = 8 mice in each group). * *p* < 0.05; ** *p* < 0.01; *** *p* < 0.001.

To study the involvement of in RBPP-P action in hepatic steatosis and insulin resistance, we examined autophagy in the liver of mice treated with normal chow, HFD, and HFD plus RBPP-P for 8 weeks by detecting p62 degradation and LC3-II conversion using Western blots. The results demonstrated that HFD suppressed autophagy and RBPP-P treatment attenuated HFD-induced inhibition in hepatic autophagy (Figure 6A).

To explore the potential mechanisms by which RBPP-P activated autophagy, we examined the signaling pathway involving AMPK and mTOR, two major upstream signaling molecules that regulate autophagy and lipid metabolism in the liver, as indicated by previous publications [12,13]. HFD induced a remarkable reduction in AMPK phosphorylation and a marked elevation in mTOR phosphorylation. RBPP-P treatment significantly attenuated HFD-induced alterations in the phosphorylation of AMPK and mTOR in liver of mice (Figure 6A). Thus, we proposed that RBPP-P induced autophagy in an AMPK/mTOR-dependent manner. 

To further confirm that autophagy induction of RBPP-P was mediated by AMPK, we analyzed the effect of RBPP-P on autophagy in AMPK knockdown cells. In mouse embryonic fibroblasts (MEF) with AMPK knockdown using AMPK subunits *prkaa1/2* shRNA, RBPP-P-induced elevation in autophagy was significantly decreased as compared with normal cells, suggesting that AMPK is indispensable for RBPP-P-mediated autophagy (Figure 6B). Taken together, these data suggested that RBPP-P enhanced autophagy through activation of the AMPK pathway.

Given the implication of autophagy in adipose tissue for obesity and diabetes [14,15], we also tested autophagy level in HFD-fed mice adipose tissue with RBPP-P treatment (Figure 6C). RBPP-P treatment inhibited autophagy level neither in gonadal WAT nor in inguinal WAT, suggesting that RBPP-P enhanced autophagy specifically in the liver. 

## 3. Discussion

The current study used HepG2-cells supplemented with high concentrations of glucose and fatty acids to mimic the influx of an excess of free fatty acids into the liver in obesity and type 2 diabetes mellitus patients, leading to insulin resistance. The results demonstrated that RBPP-P treatment significantly improved insulin sensitivity to increase glucose uptake, but not RBPP-N, indicating that pectic polysaccharide could benefit to patients with obesity-associated diabetes. In HFD-fed mice, RBPP-P treatment significantly attenuated insulin resistance as compared with normal chow diet-fed mice. Previous studies have indicated that excess nutrition administration elicited insulin resistance, consequently resulting in aberrant lipid accumulation in liver [16]. Thus, we could speculate that this attenuating effect of RBPP-P on HFD-induced insulin resistance might contribute to a protection of the liver from steatosis. 

Thus, we examined the effect of RBPP-P on HFD-induced lipid accumulation in the liver of mice. Our results demonstrated that RBPP-P treatment attenuated HFD-induced lipid accumulation in hepatic cells, and more interestingly lowered serum TG and LDL-cholesterol in mice treated with HFD. In addition to its association with insulin function, hepatic steatosis is related to lipid metabolism, including lipolysis and lipogenesis. Increasing rate of lipogenesis results in fatty acids stored in non-alcoholic fatty liver subjects [17]. However, increasing the expressions or activities of lipases reduces intrahepatic TG accumulation [18]. Therefore, we detected the effects of RBPP-P on the expressions of genes related to lipolysis and lipogenesis, demonstrating that RBPP-P specifically increased lipolysis genes (*Ppar*, *Cpt1*, and *Acox1*) expressions, but not lipogenesis genes (*Srebf1* and *Fasn*). These results suggested that the protective action of RBPP-P in HFD-induced hepatic steatosis might be mediated by elevated lipolysis via the stimulation of lipase gene expressions in hepatic cells. 

Besides lipases, reduced autophagy also regulates lipid accumulation and insulin-resistance in liver [7,13,19]. This alternative lipid degradative manner in hepatocytes mobilizes large amounts of lipids despite their low level of lipases in comparison with adipocytes [20]. Autophagy in hepatocytes is inhibited in obesity, and the dampened autophagy is mediated by down-regulated expression of Atg7, an autophagy-related protein [21]. Reversely, hepatic overexpression of *Atg7* in HFD-fed mice improved fatty liver and insulin resistance. Rapamycin—an autophagy enhancer—reduces hepatic steatosis and raises insulin sensitivity [22]. These results were consistent with the current observation that hepatic autophagy was significantly impaired in HepG2 cells treated with FG conditioned medium and in the liver of HFD-fed mice. More interestingly, RBPP-P treatment significantly attenuated the inhibited autophagy both in HepG2 cells treated with FG conditioned medium and in the liver of HFD-fed mice. These results suggested that RBPP-P-induced amelioration of hepatic steatosis and insulin resistance in HFD-fed mice might be mediated by the induction of autophagy in hepatic cells.

Autophagy is promoted by AMPK, which is a key energy sensor and regulates cellular metabolism to maintain energy homeostasis [12]. On the other hand, autophagy is inhibited by mTOR, a central cell-growth regulator that integrates growth factor and nutrient signals. Our results from the current study demonstrated that AMPK activity was diminished and mTOR activity was enhanced in liver of mice treated with HFD as compared with normal chow diet. More interestingly, RBPP-P treatment significantly attenuated the alterations in liver induced by HFD. This observation indicated that RBPP-P-induced elevation in hepatic autophagy might be mediated by both AMPK and mTOR signaling pathway.

Abnormal secretion of cytokines in adipose tissue possibly leads to insulin resistance, which ultimately causes expansion of adipose tissue mass [9,10]. Both TNF-α and IL-6 are overexpressed in obese insulin-resistant subjects, the secretion of these cytokines is interrelated. TNF-α stimulates the production of IL-6. TNF-α and IL-6 inhibit lipoprotein lipase, and TNF-α also down-regulates insulin-stimulated glucose uptake and insulin receptor signaling. RBPP-P attenuated the contents of TNF-α and IL-6 in HFD-fed mice adipose tissues, indicating that it decreased fat mass and improved insulin resistance, which may be related to the regulation of inflammation. Future work will focus on the effects of RBPP-P on adipose tissue inflammation and its relationship with the improvement of insulin resistance.

In conclusion, RBPP-P protected the liver from steatosis and insulin resistance in mice with diet-induced obesity by the stimulation of autophagy via the AMPK/mTOR-dependent signaling pathway.

## 4. Materials and Methods 

### 4.1. Mice

All animal experiments were approved by the Northeast Normal University Animal Care and Use Committee. C57BL/6J mice were housed on a 12-h light–dark cycle and fed either a normal chow diet or HFD (60% fat, D12492, Research Diets, NJ, USA).

### 4.2. Primary Hepatocytes Cell Culture

Primary hepatocytes were isolated from mice with 0.5 mg/mL type II collagenase and seeded with DMEM medium (high glucose) for 3 h on collagen-coated plate supplemented with 2% fetal bovine serum, 100 U/mL penicillin, and 100 μg/mL streptomycin. The cell was cultured at 37 °C in a humidified incubator with 5% CO_2_.

### 4.3. Bee Pollen Polysaccharides Preparation

Bee pollen from *Rosa rugosa* was provided by the Feed Research Institute Chinese Academy of Agricultural Sciences. The bee pollen polysaccharides were prepared by the protocol shown in Figure 1. Briefly, bee pollen powder was extracted with hot water, and total polysaccharide (RBPP) was precipitated by ethanol. RBPP was further fractionated by DEAE-cellulose chromatography into neutral fraction (RBPP-N) and pectic fraction (RBPP-P). 

### 4.4. Bee Pollen Polysaccharides Characterization

The sugar compositions of bee pollen polysaccharides were analyzed by HPLC, and the structural feature of RBPP-P was analyzed by FT-IR and ^13^C-NMR as described in previous publications [23].

### 4.5. High Glucose and Fatty Acids-Induced Insulin Resistance In Vitro

Human liver carcinoma cell HepG2 was cultured in low glucose (5.5 mM) DMEM medium supplemented with 10% FBS and 1% antibiotics in a 96-well plate for 24 h. Cell culture medium was changed to FG conditioned medium containing 25 mM glucose and 0.5 mM palmitic acid and oleic acid (molar ratio 1:2) with or without polysaccharides for 24 h. Subsequently, the cells were treated with 1 nM insulin for 12 h in serum-free low-glucose DMEM medium. Cells were collected for glucose uptake measured by an assay kit (Applygen, Beijing, China) using a microplate reader (-Infinite F50, Tecan, Switzerland).

### 4.6. Glucose and Insulin Tolerance Tests

Mice were fasted for 4 h in glucose tolerance tests (GTT) and 6 h in insulin tolerance tests (ITT) with free access to water, and intraperitoneally injected with D-glucose (1.5 g/kg body weight) and insulin (0.75 U/kg body weight), as described in previous publications [24]. Blood was collected from tail veins at baseline and 15, 30, 60, and 120 min after injection. The serum glucose levels were measured by the OneTouch Ultra Easy Glucometer (Johnson, New Brunswick, NJ, USA). The data was plotted as blood glucose concentrations over time.

### 4.7. Biochemical and Metabolic Parameters Analysis

The levels of serum triglyceride (TG), non-esterified free fatty acid (NEFA), cholesterol, alanine transaminase (ALT), and aspartate transaminase (AST) were measured according to the corresponding kits (Jiancheng, Nanjing, China). The level of insulin was measured using a commercially available kit (IBL international, Hamburg, Germany).

### 4.8. Quantitative Real-Time PCR (RT-qPCR)

Total RNA of the cells was extracted using Trizol (Thermo Fisher, Waltham, MA, USA) according to the instructions provided by the manufacturer. cDNA was generated from 2 μg of RNA using M-MLV reverse transcriptase (Promega, Fitchburg, WI, USA). qPCR was performed using SYBR Green Real-Time PCR Master Mixes (Thermo Fisher, Waltham, MA, USA) and LightCycler 480 real-time PCR system (Roche Applied Science, Indianapolis, IN, USA). qPCR primers are listed in Table S1.

### 4.9. Histology

The paraffin sections of liver were stained with hematoxylin and eosin (H&E) as detailed in the previous publication [18].

### 4.10. Western Blots

Cell or mouse liver extracts were prepared in lysis buffer containing 50 mM Tris (pH 7.4), 150 mM NaCl, 1 mM EDTA, 1% Triton X-100, proteinase inhibitor cocktail (Roche Applied Sciences), and phosphatase inhibitor cocktail (Thermo Scientific, Waltham, MA, USA). Western blots were performed as described in the previous publication [24] using the following primary antibodies: anti-LC3 (Novus Biologicals, Littleton, CO, USA), anti-p62 (Abnova, Taiwan, China), anti-p-insulin receptor (Cell Signaling Technology, Danvers, MA, USA), anti-p-AKT (Cell Signaling Technology, Danvers, MA, USA), anti-insulin receptor (Cell Signaling Technology), anti-AKT (Cell Signaling Technology), or anti-actin (Abcam, Cambridge, MA, USA) antibodies.

### 4.11. Statistical Analysis

The results were expressed as means ± SD. Statistical analysis of the data was performed using Student’s *t*-test and one-way ANOVA (IBM SPSS Statistics 17.0, Armonk, NY, USA). Differences were considered significant when *p* < 0.05.

## Figures and Tables

**Table 1:** Yields and monosaccharide compositions of polysaccharide fractions.

Fraction	Yield (%)	Monosaccharide Compositions (%)				
		Glc	Ara	Gal	Rha	GalA
RBPP	5.0 ^a^	14.3	48.5	22.9	3.2	11.1
RBPP-N	23.4 ^b^	35.0	37.8	27.2	--	--
RBPP-P	35.8 ^b^	2.9	51.8	21.0	5.4	18.9

^a^ Yield as % of bee pollen dry weight; ^b^ yield as % of fraction applied to column. Glc: glucose; Ara: arabinose; Gal: galactose; Rha: rhamnose; GalA: galacturonic acid.

## Supplementary Materials

The following are available online. Table S1: Primers used for qRT-PCR. Figure S1: Effects of RBPP-P on physiological activities of mice. Figure S2: Effects of RBPP-P on contents of cytokines in adipose tissues.
