# Supplementary material for: Pectic Bee Pollen Polysaccharide from Rosa rugosa Alleviates Diet-Induced Hepatic Steatosis and Insulin Resistance via Induction of AMPK/mTOR-Mediated Autophagy
Source: Molecules. 2017 Apr 28;22(5):699. doi: 10.3390/molecules22050699 (PMC6154703; doi:10.3390/molecules22050699)
Supplement: Supplementary file 1 [file molecules-22-00699-s001.pdf]

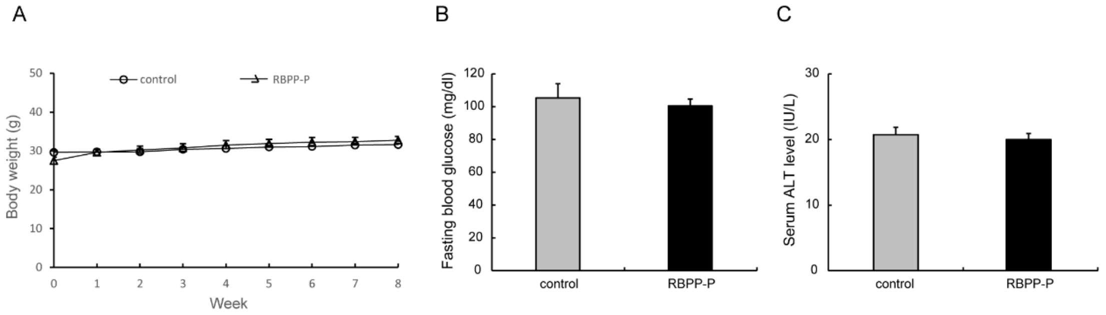

Figure S1. Effects of RBPP-P on the physiological activities of mice. Mice were treated with RBPP-P (20 mg/kg, intraperitoneal injection, once daily) for 8 weeks. (**A**) Effect of RBPP-P on body weight; (**B**) Effect of RBPP-P on fasting blood glucose; (**C**) Effect of RBPP-P on serum alanine transaminase (ALT) level. Results represent mean ± s.d. (n = 6 mice in each group).

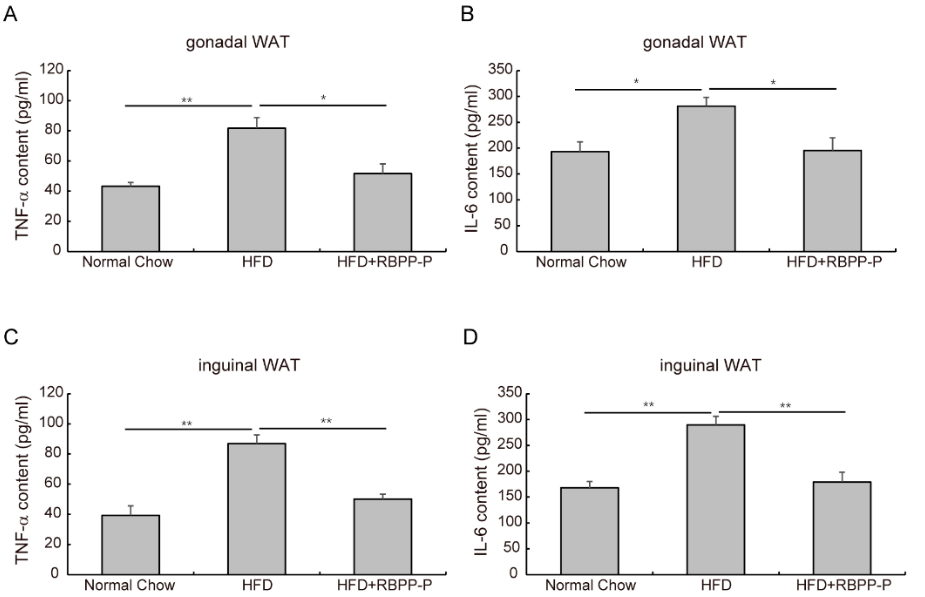

Figure S2. Effects of RBPP-P on contents of cytokines in adipose tissues. HFD-fed mice were treated with RBPP-P (20 mg/kg, intraperitoneal injection, once daily) for 8 weeks. (**A**) Tumor necrosis factor α (TNF-α) content in gonadal WAT (white adipose tissue). (**B**) Interleukin 6 (IL-6) content in gonadal WAT. (**C**) TNF-α content in inguinal WAT. (**D**) IL-6 content in inguinal WAT. Results represent mean ± s.d. (n = 8 mice in each group). * $p < 0.05$; ** $p < 0.01$.

Table S1. Primer sequences for qPCR.

Genes	Forward	Reverse
Pparα	5′-CTTCAACATGAACAAGGTCAAAGC-3′	5′-AGCCATACACAGTGTCTCCATATC-3′
Cpt1a	5′-CTCAGTGGGAGCGACTCTTCA-3′	5′-GGCCTCTGTGGTACACGACAA-3′
Acox1	5′-CAGCCAGATTGGTAGAAATTGCT-3′	5′-ACGCCACTTCCTTGCTCTTC-3′
Srebf1	5′-AACGTCACTTCCAGCTAGAC-3′	5′-CCACTAAGGTGCCTACAGAGC-3′
Fasn	5′-TTCCAAGACGAAAATGATGC-3′	5′-AATTGTGGGATCAGGAGAGC-3′
Gapdh	5′-TGTGTCCGTCGTGGATCTGA-3′	5′-TTGCTGTTGAAGTCGCAGGAG-3′
